# Mutated *Pkhd1* alone is sufficient to cause autoimmune biliary disease on the nonobese diabetic (NOD) genetic background

**DOI:** 10.1007/s00251-022-01276-3

**Published:** 2022-09-13

**Authors:** David E. Adams, Luke S. Heuer, Manuel Rojas, Weici Zhang, William M. Ridgway

**Affiliations:** 1 Division of Immunology, Allergy and Rheumatology, University of Cincinnati College of Medicine, Cincinnati, OH 45267 USA; 2 Department of Internal Medicine, Cincinnati VA Medical Center, Cincinnati, OH 45267 USA; 3 School of Medicine and Health Sciences, Doctoral Program in Biological and Biomedical Sciences, Center for Autoimmune Diseases Research (CREA), Universidad del Rosario, Bogota, Colombia; 4 Department of Internal Medicine, Sacramento VA Medical Center, VA Northern California Health Care System, Mather, CA 95655 USA; 5 Division of Rheumatology, Allergy and Clinical Immunology, University of California, Davis, CA 95616 USA

**Keywords:** NOD, *Pkhd1*, Autoimmunity, Primary biliary cholangitis (PBC)

## Abstract

We previously reported that nonobese diabetic (NOD) congenic mice (NOD.*c3c4* mice) developed an autoimmune biliary disease (ABD) with similarities to human primary biliary cholangitis (PBC), including anti-mitochondrial antibodies and organ-specific biliary lymphocytic infiltrates. We narrowed the possible contributory regions in a novel NOD.*Abd3* congenic mouse to a B10 congenic region on chromosome 1 (“*Abd3*”) and a mutated *Pkhd1* gene (*Pkhd1*^del36−67^) upstream from *Abd3*, and we showed via backcrossing studies that the NOD genetic background was necessary for disease. Here, we show that NOD.*Abd3* mice develop anti-PDC-E2 autoantibodies at high levels, and that placing the chromosome 1 interval onto a *scid* background eliminates disease, demonstrating the critical role of the adaptive immune system in pathogenesis. While the NOD genetic background is essential for disease, it was still unclear which of the two regions in the *Abd3* locus were necessary and sufficient for disease. Here, using a classic recombinant breeding approach, we prove that the mutated *Pkhd1*^del36−67^ alone, on the NOD background, causes ABD. Further characterization of the mutant sequence demonstrated that the *Pkhd1* gene is disrupted by an ETnII-beta retrotransposon inserted in intron 35 in an anti-sense orientation. Homozygous *Pkhd1* mutations significantly affect viability, with the offspring skewed away from a Mendelian distribution towards NOD *Pkhd1* homozygous or heterozygous genotypes. Cell-specific abnormalities, on a susceptible genetic background, can therefore induce an organ-specific autoimmunity directed to the affected cells. Future work will aim to characterize how mutant *Pkhd1* can cause such an autoimmune response.

## Introduction

Primary biliary cholangitis (PBC) is an incurable autoimmune disease characterized by highly specific antimitochondrial autoantibodies (AMAs) and autoimmunity directed to cholangiocytes, with progressive destruction of intrahepatic bile ducts. Some progress has been made in understanding human PBC, including identifying the target mitochondrial autoantigens which induce the characteristic anti-pyruvate dehydrogenase complex (anti-PDC-E2) autoantibodies, mapping the T and B cell autoantigenic epitopes, and defining mechanisms of tissue destruction (Lleo et al. 2020). We serendipitously discovered a model of PBC while studying type 1 diabetes (T1D) in nonobese diabetic (NOD) mice (Irie et al. 2006a, b; Koarada et al. 2004). We found that NOD congenic mice with B6/B10 regions inserted onto the NOD background (strain NOD.*c3c4*) were protected from diabetes but developed a spontaneous autoimmune biliary disease. Lymphoid cell infiltrates in these mice specifically targeted the biliary epithelium, not the hepatic parenchyma, and demonstrated classic histological lesions of PBC including granulomas, peri-biliary lymphocytic infiltration, and nonsuppurative destructive cholangitis (NSDC), considered highly characteristic of PBC. Importantly, there was a highly specific immune signature identical between NOD.*c3c4* mice and human PBC: the strain was the first ever to spontaneously develop anti-PDC-E2 antibodies, the hallmark of human PBC (Irie et al. 2006a, b). The NOD.*c3c4* model thus bore many remarkable similarities to human PBC although, like most mouse models, this model has some differences from human PBC. Therefore, we term the model “autoimmune biliary disease” (ABD). However, the presence of anti-PDC-E2 antibodies, the characteristic immunopathological lesions, and the involvement of the immune system are strong reasons to use these mice to model pathogenic events in PBC.

We used a mouse congenic approach to study the immunogenetic causes of NOD ABD (Wicker et al. 1995). We developed a large number of congenic mice, termed “NOD.ABD” mice overall, with the initial goal of narrowing the congenic chromosome 3 and 4 (c3, c4) intervals to find causative genes. During this process, a 5 k SNP chip detected a heretofore unrecognized region on chromosome 1 in all the NOD.ABD strains that developed disease; we designated this region *Abd3* (Yang et al. 2011). Subsequently, we made a congenic mouse (“NOD.*Abd3*”) expressing only the *Abd3* region on the NOD background and showed that this c1 region is necessary and sufficient for disease. We made a major breakthrough in understanding how this region causes disease, using RNA-seq to show that *Pkhd1* (Polycystic Kidney and Hepatic Disease 1), which is upstream on chromosome 1, is mutated in NOD.*Abd3* mice with truncation after exon 35 (designated “*Pkhd1*^*del36*−67^”) (Huang et al. 2018). *Pkhd1* encodes fibrocystin, a large protein that is expressed in primary cilia including cholangiocyte cilia (Nagasawa et al. 2002; Ward et al. 2003). Mutations in *Pkhd1* can result in either renal, pancreatic, or biliary disease, manifested by cyst formation (Gallagher et al. 2008; Moser et al. 2005; Williams et al. 2008). Some *Pkhd1* mutations, including *Pkhd1*^*del36*−67^ and *Pkhd1*^del4/del4^, however, result in liver-specific disease; in the *Pkhd1*^del4/del4^ model, this disease was ameliorated by macrophage depletion, showing a role of the immune system in pathogenesis (Locatelli et al. 2016). In the NOD.*Abd3* mice, the NOD genetic background was essential for the disease; in addition, adaptive immunity was necessary (since NOD.c3c4-*scid* mice did not develop clinical disease) (Huang et al. 2018). However, it was still unclear which genetic regions in the *Abd3* interval contributed to the disease: The B6 congenic interval (termed “B6-*Abd3*”) could play a role in conjunction with *Pkhd1*^*del36*−67^ to mediate autoimmunity in this model. Herein, we show that the NOD.*Abd3* mouse, like the original NOD.*c3c4* mouse, develops high levels of the PBC-specific anti-PDC-E2 autoantibodies. In addition, we show that NOD.*Abd3* mice require an adaptive immune system to develop the disease. Finally, using a classic recombinant breeding approach, we prove that the mutated *Pkhd1*^del36−67^ alone, on the NOD background, causes ABD in this strain. Cell-specific abnormalities (in this case, aberrant *Pkhd1* expression in cholangiocytes) on a susceptible genetic background can therefore induce an organ-specific autoimmunity directed to the affected cells.

## Materials and methods

### Processing of tissues for PCR

Genomic DNA from NOD and NOD.*Abd3*-derived tissues (e.g., ear, tail) was prepared using QIAGEN’s DNeasy Blood & Tissue kit. The isolated DNAs were stored at − 20 °C until use. The DNA primers used for genotyping of the NOD and NOD.*Abd3* mice and offspring were designed using either Primer3 or PCRTiler v1.42 software. The DNA primers were purchased from Sigma-Aldrich.

### *Abd3* PCR

To distinguish between the NOD and NOD.*Abd3* alleles, both PCR amplification and restriction digestion are required. The sequences of the four primers used for Abd3 genotyping are the following:

- Name:                  rs32160775-F
- Sequence:            5′ AGATCTCGGGTGTGGAGTCA 3′
- Name:                  rs32160775-R
- Sequence:            5′ CCACTTCCCAAAGTGGTCAT 3′
- Name:                  rs36742082-F
- Sequence:            5′ AAAGGCAGTGTGCTCAAGGT 3′
- Name:                  rs36742082-R
- Sequence:            5′ TCTGAGGTGGATCTGGAAGC 3′

A DNA Engine Thermal Cycler (MJ Research) was used for PCR amplification. The program used included the following steps:

- 95 °C for 15:00                      (i.e., 15 min)*
- 95 °C for 0:10                        (i.e., denature for 10 s)
- 55 °C for 0:30                        (i.e., anneal for 30 s)
- 72 °C for 0:30                        (i.e., extend for 30 s)
- GOTO Step 2 for 35 times     (i.e., cycle 35 times)
- 72 °C for 5:00                       (i.e., 5 min final extension)
- 4 °C for 0:00                         (i.e., 4 °C indefinite)
- END

AmpliTaq Gold DNA polymerase (Applied Biosystems, Cat. No. N808-0241) was purchased to amplify the genomic DNA. This polymerase is supplied in an inactive state and must be activated by heat, either before or during PCR cycling. *Step 1 of the PCR protocol accomplishes this. After DNA amplification, the individual PCR products were divided in two, and one half of each of the 112-bp rs32160775 amplification products was restricted with BmrI at 37 °C for 1 h, while half of each of the 121-bp rs36742082 products was restricted with BsmAI at 55 °C for 1 h. The NOD rs32160775 products are cleaved by BsmAI, while the NOD.*Abd3* products are not. The NOD.*Abd3* rs36742082 products are cleaved by BsmAI, while the NOD products are not. The two sets of reactions and PCRs thus give complimentary genotyping data. The final DNA products were run on a 3.0% agarose gel to distinguish the DNA products based on their size. Restriction endonucleases were purchased from New England BioLabs and UltraPure™ Agarose from Invitrogen (Cat. No. 16500–100). The electrophoresed DNAs were stained using 0.05 μg/ml ethidium bromide for 30 min, then the gels rinsed with water to remove background staining, and the PCR products visualized by high-intensity, 302-nm UV light photography using a Cell Biosciences, Inc.’s FluorChem HD2 gel doc system with AlphaView version 3.2.2.0 software. Gel images were opened in Adobe Photoshop CC 2018 and exported to PowerPoint 2016 for processing.

### *Pkhd1* PCR

The sequences of the four primers used to genotype the NOD and NOD.*Abd3 Pkhd1* alleles are as follows:

- Name:                 P7f
- Sequence:           5′ TGGATAACTTCATCCCTCCTTAAA 3′
- Name:                 P1r
- Sequence:           5′ TGGTAGCCACAGATGGCTTT 3′
- Name:                 P2f
- Sequence:           5′ AACCCCTAATTCCCAGGTCA 3′
- Name:                 P2r
- Sequence:           5′ TGAGCACGTGCATGCTATGA 3′

The *Pkhd1* allele PCR amplification program used was the same as that used for the Abd3 PCR. After cycling program was finished, the amplified PCR products were run on a 2.0% agarose gel to distinguish the products based on their size. The “wild type” NOD *Pkhd1* intron 35 P2f + P2r PCR yields a 313-bp product, while the “mutant” NOD.*Abd3 Pkhd1* intron 35 P7f + P1r PCR yields a 379-bp product. Occasionally, additional amplification products are seen, especially using the P7f + P1r primers, but the source of these larger ~ 700-bp DNA products is unknown.

### Identification of ETnII-beta retrotransposon integrant in the NOD.*Abd3 Pkhd1* allele

A retrotransposon integrant in the NOD.*Abd3 Pkhd1* allele was originally identified by (Huang et al. 2018). Further characterization of the integrant and its PCR genotyping were performed here. Node-1, node-2, and node-3 sequences (see Fig. 8E (Huang et al. 2018)) originally obtained by sequencing the NOD and NOD.*Abd3* mice Chr. 1 *Pkhd1* intron 35 regions were entered into BLASTn. Extensive homology of the node sequences was found to the 5541-bp *Mus musculus* retrotransposon ETnII-beta, complete sequence (GenBank: KC12757.1). These five sequences, i.e., the complete ETnII-beta sequence, the relevant NOD *Pkhd1* intron 35 region, and the NOD.*Abd3 Pkhd1* node-1, node-2, and node-3 regions, were then entered into the CLUSTAL O (1.2.4) alignment program, and the resulting alignment further refined by visual analysis. Two CTTTCC repeats were identified that flank the integrated ETnII sequence in the NOD.*Abd3 Pkhd1* intron 35 mutant allele, with a single CTTTCC site in the original NOD sequence marking the retrotransposon integration site. Except for a single TT pair located at position 2996–97 in the KC12757.1 sequence and missing in the mutant *Pkhd1* sequence, and an extra TT pair present in the mutant *Pkhd1* sequence at position 5200 in the PubMed ETnII sequence, the ETnII-beta integrant in the mutant *Pkhd1* intron 35 is essentially complete. Binding sites for the *Pkhd1* genotyping primers P2r, P1r, P7f, and P2f were identified in the NOD.*Abd3 Pkhd1* sequence with the predicted products of PCR agreeing with what was observed (see gel figures and mutant allele diagram).

### Animal husbandry and histological scoring

Animals were housed in the Cincinnati VA Animal facility and provided food and water ad libitum. Regular feeding chow was used, even for breeder pairs. Tissue samples, obtained from either the ear or tail, were typically taken when the pups were weaned at 3 weeks of age and again at sacrifice. The mice were observed for phenotype from birth and processed for histology between 4 and 5 months of age, unless they showed signs of liver disease earlier. Mice for breeding pairs were identified by PCR to separate the closely linked NOD- and NOD.*Abd3*-derived *Pkhd1* and Abd3 alleles. Liver samples for histology were stored in fixative (10% buffered formalin, Fisher Health Care) until embedded in paraffin, sectioned for H&E staining, and digitally imaged by the Cincinnati Children’s (CCHMC) Pathology Research Core. At the time of sacrifice, liver appearance and common biliary duct (CBD) diameter were scored for the NOD- and NOD.*Abd3*-derived animals. Liver sections were scored for biliary disease using the following scale: (0) No bile duct disease; (1) Mild bile duct disease: at least 25% of bile ducts affected by inflammatory infiltrates; (2) Moderate bile duct disease: at least 50% of bile ducts affected; (3) Severe bile duct disease: at least 75% of bile ducts affected. Gross pathology of the liver was scored as follows: “0” normal; “1” very few lesions in the liver detectable; “2” lesions in liver easily noticed; “3” many liver lesions easily seen; “4” many lesions in most or all lobes; “5” liver completely full of lesions.

### Generation of recombinant mice and breeding *Pkhd1*^mu/mu^ homozygosity

NOD and NOD.*Abd3* mice we bred to generate F1 mice, which were crossed to generate F2 mice. F2 mice were screened to identify recombinant mice (between the Abd3 and *Pkhd1* loci), which were either homozygous for the NOD *Abd3* allele (i.e., were not congenic in the *Abd3* region) but heterozygous at *Pkhd1*, or heterozygous at the *Abd3* locus but homozygous for *Pkhd1*^mu^ (either genotype indicated a recombinant event between the loci). Four recombinant mice (3 males and 1 female) were identified out of 73 genotyped F2 mice. The recombinant male F2 breeders were backcrossed to NOD mice to produce F3 mice that were homozygous for NOD *Abd3* and heterozygous at *Pkhd1*. These were intercrossed to produce F4 mice that were genotyped and phenotyped to detect mice with homozygosity for *Pkhd1*^*del36*−67^. Additional F5 mice were generated by F4 intercrosses and also genotyped/phenotyped.

### Detection of serum AMA levels

Recombinant pyruvate dehydrogenase complex-E2 (PDC-E2) protein was used to detect serum levels of anti-mitochondrial antibodies by enzyme-linked immunosorbent assay (ELISA) using well-established protocols from our laboratory (Bae et al. 2016; Tsuda et al. 2013). PDC-E2 recombinant protein at a concentration of 5 μg/ml in carbonate buffer (pH 9.6) was coated on 96-well ELISA plates at 4 °C overnight. Plates were then washed 3 × with phosphate-buffered saline Tween-20 (PBS-T 0.05%) prior to incubation with a blocking solution of 3% non-fat dry milk in PBS for 30 min at room temp (RT). Serum samples diluted 1:250 in blocking solution were then incubated in wells for 1 h at RT, followed by washing 3 × with PBS-T. A mix of horseradish peroxidase (HRP)–conjugated anti-human immunoglobulin (A + M + G, heavy and light chain) (Invitrogen, Carlsbad, CA) at a 1:2000 dilution was added for 1 h at RT and then washed 3 × with PBS-T. TMB peroxidase substrate (BD Biosciences, San Jose, CA) was then added to wells, incubated for 15 min at RT, and then read at OD_450nm_ on a microplate reader. Control serum samples for both positive and negative AMA levels were included on each plate.

## Results

**Fig. 1:**
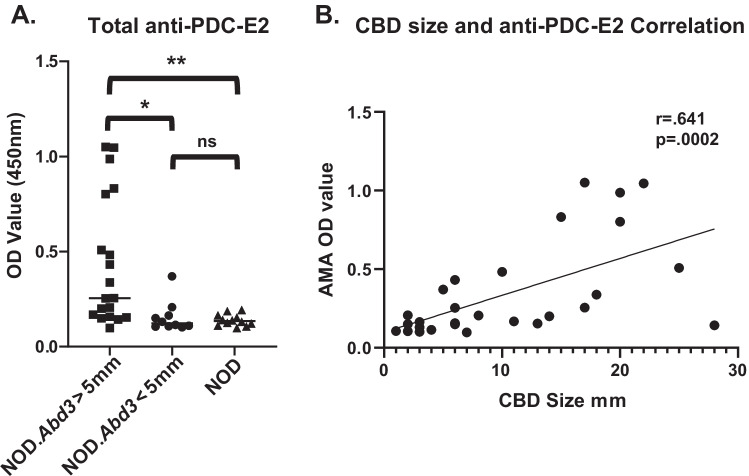
Elevated serum level of autoantibody against mitochondrial antigen PDC-E2 was detected in NOD.*Abd3* mice as compared to NOD control. **A** Serum autoantibody levels against PDC-E2 were measured using ELISA. **B** The serum autoantibody level positively correlated with common bile duct size by Spearman correlation *r* = 0.641 *p* = 0.0002. NOD.*Abd3* > 5 mm (*n* = 19); NOD.*Abd3* < 5 mm (*n* = 10); NOD mice (*n* = 12); *p < 0.05; **p < 0.01 by ANOVA with Tukey’s post hoc test

**Fig. 2:**
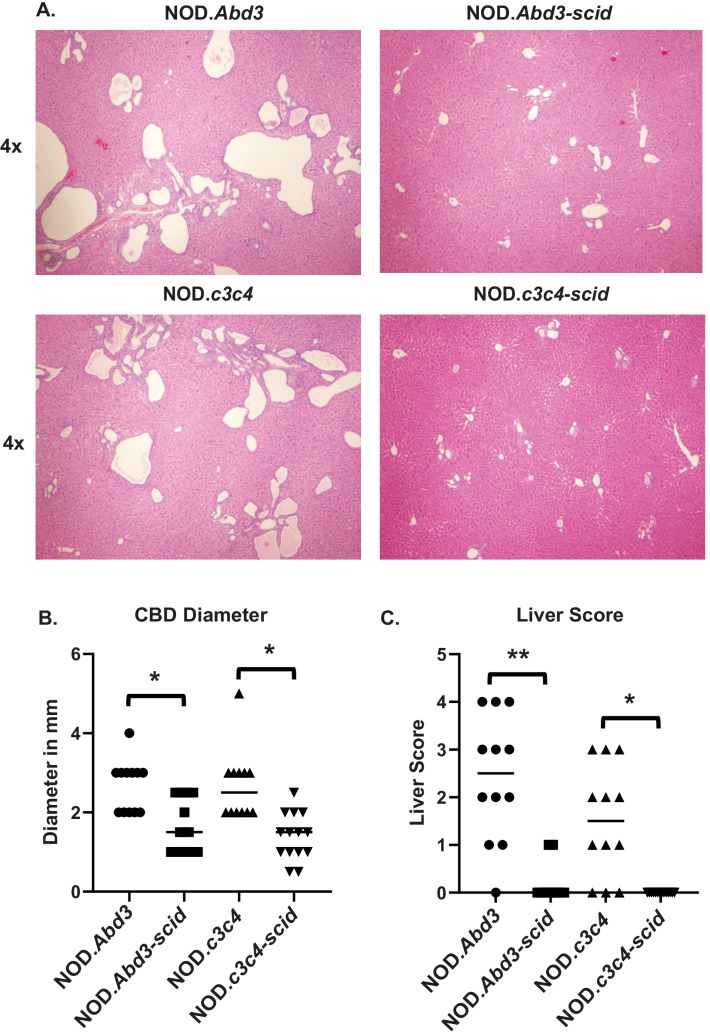
NOD.*Abd3* mice lacking the B6.*c3c4* congenic intervals present with the same histopathological abnormalities as the NOD.*c3c4* strain. **A** H + E stained liver sections from 120- to 130-day NOD.*Abd3* and NOD.*c3c4* (left) with respective *scid* mutations (right) showing comparable histology and lack thereof in the absence of adaptive immune cells; 4 × magnification. **B** Dilation of the common bile duct (in mm) in NOD.*Abd3* and NOD.*c3c4* with significant reduction in the absence of adaptive immunity (*scid*). **C** Gross liver pathology scoring of disease in NOD.*Abd3* and NOD.*c3c4* with significant reduction on a *scid* background. *p < 0.01, **p < 0.001 by Kruskal–Wallis with Dun’s post hoc test. NOD.*Abd3* (*n* = 12), NOD.*Abd3*-*scid* (*n* = 16), NOD.*c3c4* (*n* = 12), NOD.*c3c4-scid* (*n* = 14)

The original NOD.*c3c4* mouse had multiple B6/B10 congenic intervals that had a profound effect on NOD immunity: these regions completely suppressed development of type 1 diabetes in this strain (Irie et al. 2006a, b; Koarada et al. 2004). In particular, the *Idd9.3* region was responsible for development of autoantibodies such as ANAs and anti-Smith autoantibodies (Irie et al. 2006). We therefore tested whether NOD.*Abd3* mice developed the signature anti-PDC-E2 autoantibodies found in the NOD.*c3c4* strain, despite lacking the c3 and c4 congenic regions. As shown in Fig. 1, NOD.*Abd3* mice developed high titers of anti-PDC-E2 autoantibodies. Common bile duct (CBD) dilation is a pathological marker of clinical disease development in these mice (Irie et al. 2006a, b; Koarada et al. 2004), and NOD.*Abd3* mice without CBD dilation had significantly lower anti-PDC-E2 antibody levels (Fig. 1a). Increased CBD size correlates with worse disease (Irie et al. 2006a, b; Koarada et al. 2004), and anti-PDC-E2 antibody levels significantly correlate with CBD size (Fig. 1b). Likewise, NOD.*Abd3* mice develop comparable CBD diameter dilation to NOD.*c3c4* mice (Fig. 2b) and gross pathological abnormalities in the liver as measured by cyst burden (Fig. 2C). Furthermore, we have previously shown that the adaptive immune system was necessary for ABD, since NOD.*c3c4*-*scid* mice did not develop clinical disease, but only minor histological abnormalities (Huang et al. 2018; Irie et al. 2006a, b; Yang et al. 2011). We next confirmed that mice expressing only the Abd3 region on the NOD background did not develop clinical disease when the *scid* mutation was introduced (Fig. 2). Thus, the *Abd3* region alone was sufficient to develop biliary autoimmunity and PBC autoantibodies on the NOD background. The major features of autoimmune biliary disease were therefore found to remain in this strain with only the minimal Abd3 congenic interval. However, it was still unclear whether the B6-Abd3 region, the mutated *Pkhd1* region, or both were necessary for disease.

**Fig. 3:**
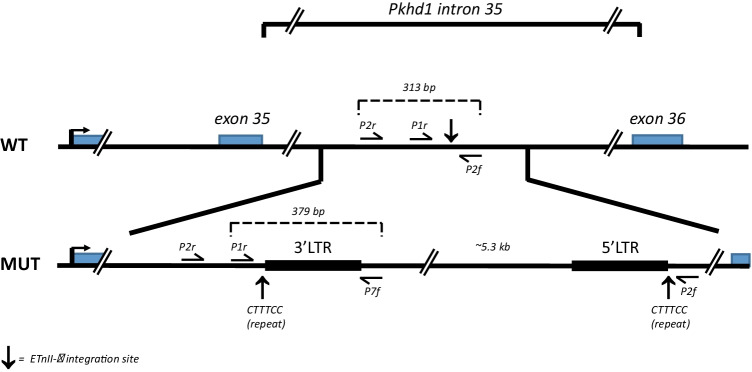
Schematic of retrotransposon insertion site into intron 35 of *Pkhd1*. The ETnII-beta retrotransposon in the NOD.*Abd3* mouse is inserted into the *Pkhd1* gene in the antisense orientation

**Fig. 4:**
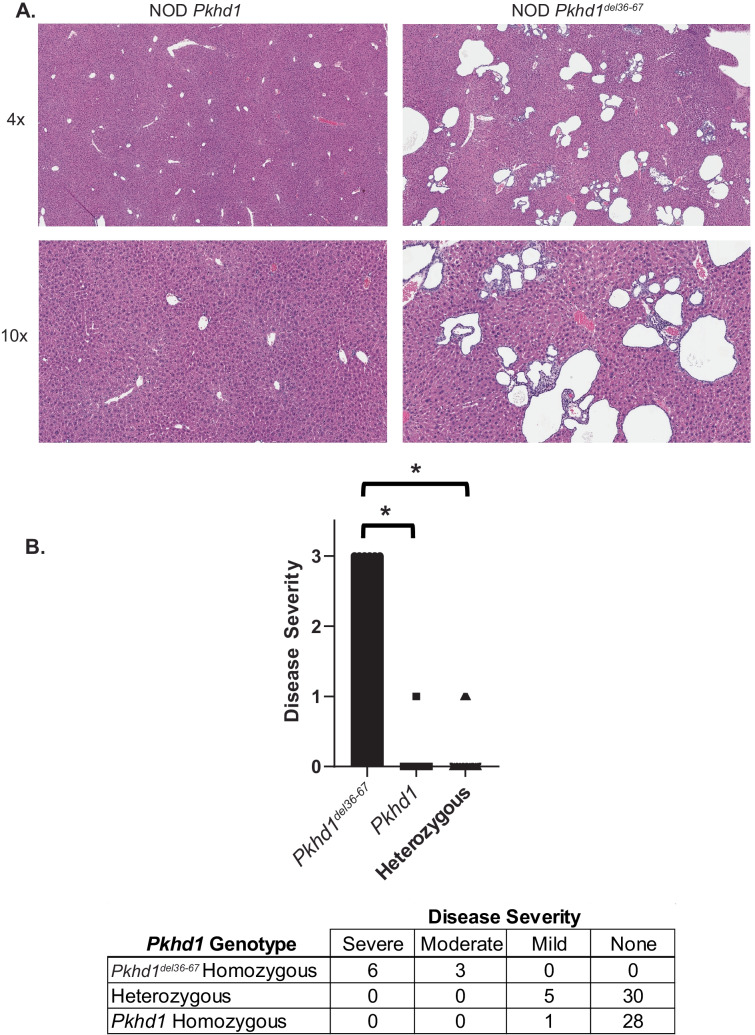
Histological abnormalities persist in F4 mice lacking the B6.Abd3 congenic interval but homozygous for the *Pkhd1*^*del36*−67^ mutation. **A** H + E stained liver sections showing immune infiltration and bile duct dilation only in those mice homozygous for *Pkhd1*^*del36*−67^; 4 × and 10 × magnification. **B** Histological scoring of liver H + E sections; *p < 0.01 by Fisher’s exact test

Recombinant mice were generated as described (see “Materials and methods”), and mice were bred by further backcrossing to generate mice that were heterozygous or homozygous for *Pkhd1*^*del36*−67^ and which lacked the B6-Abd3 congenic interval. Generation of PCR primers to detect the *Pkhd1*^*del36*−67^ mutation revealed that the ETnII-beta retrotransposon causing the mutation is inserted into the *Pkhd1* gene in the antisense orientation (“Materials and methods”, Fig. 3), which has possible implications for pathological effects (see “Discussion”). Fifty-five F4 and twenty-one F5 mice were generated (Table 1). Of these, the NOD (wild type) *Pkhd1* and heterozygous mice were in numbers as expected by a Mendelian distribution; however, the number of *Pkhd1*^*del36*−67^ homozygous mice was significantly reduced (Table 1), which might reflect decreased viability (see “Discussion”). Figure 4a shows two littermates; the mouse with homozygous *Pkhd1*^*del36*−67^ demonstrates the characteristic findings of biliary epithelial proliferation with immune cell infiltrates adjacent to cholangiocytes (Fig. 4a), identical to the histological picture in NOD.*c3c4* and NOD.ABD mice as previously described (Irie et al. 2006a, b; Koarada et al. 2004; Yang et al. 2011) and as shown in Fig. 2a. The offspring with wild-type *Pkhd1* displayed no abnormalities (Fig. 4a). Blinded scoring of the histology in these mice very clearly showed that ABD is present in mice lacking the B6-Abd3 congenic interval but only in mice with two copies of *Pkhd1*^*del36*−67^ (Fig. 4). This result proves definitively that the *Pkhd1*^*del36*−67^ mutation is the only genetic component in the Abd3 region necessary to promote ABD on the NOD genetic background.

**Table 1:** Genotype distribution of NOD vs. mutated *Pkhd1*. Expected numbers according to a Mendelian distribution are in parentheses

	NOD *Pkhd1*	Heterozygous	Mutated *Pkhd1*
F4	15 (14)	34 (28)	6 (14)
F5	7 (5)	12 (11)	2 (5)

## Discussion

The NOD mouse is best known as a model of spontaneous T1D; however, they develop multiple other spontaneous autoimmune conditions such as thyroiditis and Sjögren’s syndrome. Immunogenetic components contributory to NOD autoimmune disease include the TCR-MHC interaction (signal 1), co-stimulatory molecules (signal 2), and cytokines (signal 3) as reviewed by Aubin et al. (Aubin et al. 2022). The NOD immunogenetic background is susceptible to autoimmunity and to understand the basis of this heightened immunity, congenic mapping experiments were conducted to identify genetic regions responsible for the diabetes phenotype. This approach identified multiple genes and genetic regions (over 50 *Idd* regions to date (Driver et al. 2012)). The autoimmune biliary disease (ABD) phenotype emerged during congenic experiments designed to completely eliminate T1D by introgressing large congenic regions from chromosomes 3 and 4 onto the NOD background (the NOD.*c3c4* mice). Initially, this was a perplexing result as it was unclear how introducing non-NOD alleles could protect against diabetes but simultaneously cause the NOD mouse to switch to autoimmune liver specificity (Ridgway 2006). The original hypothesis was that immune-related genes within the chromosome 3 or 4 region might control organ specificity. However, we subsequently discovered a small non-NOD genetic region on chromosome 1 that was present in all strains afflicted with disease (Huang et al. 2018). Further investigation using RNAseq found that the mRNA of a non-immune-related gene *Pkhd1* had been truncated by a retrotransposon insertion. To ensure that the non-NOD B6-Abd3 region was not responsible for the autoimmune phenotype, herein we created recombinant mice lacking the B6-Abd3 region and only expressing the mutated *Pkhd1* on the NOD background. Therefore, for the first time, we have shown that a non-immune, tissue-specific mutation on the hyperimmune NOD background can promote an organ-specific autoimmune disease.

The *Pkhd1* gene encodes fibrocystin/polyductin, a large single-membrane spanning protein of unknown function (Bergmann 2018). It is widely expressed during fetal development and thought to be involved in tubular morphogenesis. In adulthood, expression is localized to apical membranes and the primary cilia of epithelial cells in the kidney, liver, and pancreas (Zhang et al. 2004). Aberrant expression during fetal development and its involvement in tubule morphogenesis may explain the deficiency we observe in the number of homozygous mutant pups in Table 1. Alternatively, early transposon (ETn) elements are transcribed during early mouse embryogenesis in embryonic stem (ES) and embryonic carcinoma (EC) cell lines (Maksakova and Mager 2005). Formation of an RNA:RNA hybrid between the (reverse transcribed) ETn-*Pkhd1* insert and the (forward transcribed) mutant *Pkhd1* mRNA (with the retroviral insert in intron 35) could occur during mouse embryogenesis in the mutant *Pkhd1* mouse. This could play a role in the NOD.*Abd3* model and in poor survival of neonatal homozygotes. While fibrocystin’s exact function is unknown, it is thought to interact with other receptors (polycystins) on epithelial cilia that participate in sensing of bile flow (Kim et al. 2008a, b; Olson et al. 2019). In the PCK rat, mutations in *Pkhd1* result in loss of fibrocystin localization to the cilia and shorter cilia structure (Masyuk et al. 2003). These changes in cilia structure are thought to initiate a “ductal reaction” that stimulates cholangiocyte proliferation, malformation of the ductal plate, and formation of cysts. How a retrotransposon insertion in a protein involved in cilia structure and/or function can lead to an autoimmune disease in the NOD mouse is a perplexing question.

In addition to the PCK rat, several mouse models of *Pkhd1* gene disruption have been produced to model human autosomal recessive polycystic kidney disease (ARPKD) and congenital hepatic fibrosis (CHF). These include *Pkhd1*^*lacZ/lacZ*^ (exon 1–3) (Williams et al. 2008), *Pkhd1*^*del2/del2*^ (exon 2) (Woollard et al. 2007), *Pkhd1*^*ex40*^ (exon 40) (Moser et al. 2005), *Pkhd1*^*del3−4/del3−4*^ (exon 3–4) (Garcia-Gonzalez et al. 2007), *Pkhd1*^*del4/del4*^ (exon 4) (Gallagher et al. 2008), *Pkhd1*^C642*/ C642*^ (exon 20) (Shan et al. 2019), *Pkhd1*^*LSL(*−*)/LSL(*−*)*^ (exon 3)(Bakeberg et al. 2011), and *Pkhd1*^*e15GFPΔ16*^ (exon 15–16) (Kim et al. 2008a, b). Depending on the site of the *Pkhd1* gene disruption and the genetic background of the mouse, the affected tissue and severity can vary tremendously. For example, the *Pkhd1*^*lacZ/lacZ*^, *Pkhd1*^*del3−4/del3−4*^, *Pkhd1*^C642*/ C642*^, *Pkhd1*^*e15GFPΔ16*^, and *Pkhd1*^*del2/del2*^ all develop renal cysts whereas *Pkhd1*^*ex40*^ and *Pkhd1*^*del4/del*^ do not. Interestingly, when the *Pkhd1*^*del2/del2*^ strain was inbred back to either C57BL/6 J or BALBc/J, cysts developed much earlier in life and disease was more severe. Pancreatic cysts of varying severity are also found in all but the *Pkhd1*^*ex40*^ and *Pkhd1*^C642*/ C642*^ strains. The *Pkhd1*^*e15GFPΔ16*^ strain was reported to occasionally develop brain cysts and abnormal ulcers in the gastrointestinal tract. The most consistent pathologic feature is cyst development of the intrahepatic bile ducts along with fibrosis of the periportal tract.

The most striking difference between these eight models and our NOD *Pkhd1* mutant model is that, prior to the Locatelli paper in 2016, none of these models described immune infiltrates, whereas immune infiltration is a major component of our model. Gallagher et al. studied the same *Pkhd1*^*del4/del4*^ model as Locatelli, but did not describe infiltrates. The pathological role of the immune system in cystic/fibrotic liver disease has thus been recognized only very recently. Locatelli et al. showed that the immune system is involved in perpetuating cysts/fibrosis in the *Pkhd1*^*del4/del4*^ mouse on the C57BL/6 background (Locatelli et al. 2016). In their model, on the non-autoimmune B6 background, dysfunctional fibrocystin causes an immune infiltrate composed largely of macrophages, resulting in upregulation of chemokines and skewing to an M2 phenotype that promotes cyst formation and peribiliary fibrosis. Elimination of macrophages by clodronate liposomes prevents this pathology, indicating that the underlying *Pkhd1*^del4/del4^ mutation can initiate a damage response, but that the innate immune response to tissue stress ultimately drives the disease. In our model, in contrast to all other models, immune infiltrates are a major component of the disease, and are not limited to macrophages but include adaptive immune cells. In our model on the autoimmune NOD background, suppression of the adaptive immune system by either irradiation (Koarada et al. 2004), anti-CD3 antibodies, or introduction of the *scid* mutation eliminated clinical disease and resulted in only minor, subclinical duct abnormalities (Irie et al. 2006a, b). While both our model and the *Pkhd1*^*del4/del4*^ model show a critical role for the immune system, another major difference between them is the time course of disease. In the *Pkhd1*^*del4/del4*^ model, disease progresses slowly, with immune infiltration at 6 months of age and mice surviving past 12 months. Our NOD *Pkhd1* models on the autoimmune-prone NOD background on the other hand develop immune infiltration by 10 weeks and the disease is often fatal by 4 months. The adaptive immune system drives this accelerated pathology via involvement of autoreactive T and B cells on the NOD background. We have previously shown that adoptive transfer of splenocytes or CD8-positive T cells alone can drive the disease (Irie et al. 2006a, b; Koarada et al. 2004; Yang et al. 2011). In addition, these mice develop class switched anti-PDC-E2 autoantibodies, indicating that T helper cells are actively driving the immune response. In our NOD models, macrophages infiltrate the lesions and undoubtedly also play a role in disease pathology, but with the addition of autoreactive T cell help, they can be fully licensed to maximize effector function. In this case, not only do we observe accelerated cyst formation but we also find non-suppurative destructive cholangitis, epithelioid granulomas, and anti-PDC-E2 antibodies that are hallmarks of PBC. In the current study, we show that the liver disease and immune infiltrates are both fully present in the NOD *Pkhd1*^del36−67^ mice lacking the B10 *Abd3* genetic region. While we have not tested this strain for anti-PDC-E2 antibodies, all the evidence suggests the NOD background drives the autoimmune component including the broad immune infiltrates seen here. However, it remains possible that the *Abd3* region contributes to antibody formation and this has not been formally excluded, which is a limitation of our study. In contrast, the *Pkhd1*^*del4/del4*^ mutation on the C57BL6 background presumably lacks autoreactive T cells and dysfunctional fibrocystin results in a classic tissue damage response from innate immune cells resulting in slowly progressive fibrosis. While both models lack a functional fibrocystin and presumably have similar tissue stress, the differing immune system response to that stress/damage controls the pathological outcome.

We have shown that the *Pkhd1*^*del36*−67^ mutation alone is the initiating factor in the observed cholangiocyte pathology. How this single mutation leads to PBC-like autoimmunity is important since the mechanism for breaking tolerance to PDC-E2 and the initiation of PBC is poorly understood. One potential mechanism is that changes in cilia structure or how the cholangiocyte senses and reacts to bile acids may lead to cellular damage and presentation of PDC-E2 to T cells (Kim et al. 2008a, b). Additionally, alterations in bile acid composition or flow can modulate microbiome composition and/or enhance susceptibility to infections via the gut. Infections, and *Escherichia coli* in particular, have been implicated in PBC etiology, likely by inducing molecular mimicry between microbial PDC-E2 and human PDC-E2 (Yang et al. 2022). Finally, the antisense insertion of a retrotransposon as the mutagenic factor may have immune-stimulating properties. Expression of the antisense retrotransposon can lead to dsRNA, a potent stimulator of an antiviral type I IFN response which, if chronically activated, can enhance autoimmune diseases (Gazquez-Gutierrez et al. 2021). In addition, early transcription of the ETnII insert during embryogenesis may help accelerate development of disease. The NOD.*Abd3* mouse therefore presents a compelling model in which to study the initiation of PDC-E2-mediated autoimmunity to better understand the pathogenesis of human PBC.
